# Seeding Activity-Based Detection Uncovers the Different Release Mechanisms of Seed-Competent Tau Versus Inert Tau via Lysosomal Exocytosis

**DOI:** 10.3389/fnins.2019.01258

**Published:** 2019-11-21

**Authors:** Yuki Tanaka, Kaoru Yamada, Kyoko Satake, Itaru Nishida, Matthias Heuberger, Tomoki Kuwahara, Takeshi Iwatsubo

**Affiliations:** ^1^Department of Neuropathology, Graduate School of Medicine, The University of Tokyo, Tokyo, Japan; ^2^Department of Biology, ETH Zürich, Zurich, Switzerland

**Keywords:** tau, propagation, seed, Alzheimer’s disease, secretion, lysosome

## Abstract

The pathological aggregation of tau characterizes a set of neurodegenerative diseases collectively referred to as tauopathies. Recent studies using cellular and animal models have suggested that tau pathology progresses by *trans*-cellular propagation. The process of propagation is mediated by certain species of extracellular tau, which are taken up by recipient cells and serve as a seed for tau aggregation. Tau propagation is currently one of the most active areas of research in dementia. Previous efforts to identify the specific tau molecules involved in propagation have suggested that multiple forms of tau with different molecular weights derived from recombinant tau or brain lysates exert seeding activity. Nonetheless, the molecular characteristics of the “extracellular” seed-competent tau as well as its release mechanisms remain to be elucidated. Given that tau is physiologically released into the extracellular space, it is critical to distinguish seed-competent tau from normal monomeric tau. Utilizing biosensor cells expressing P301S mutant tau fused to CFP/YFP, here we discriminated between seed-competent tau and inert monomer tau released from HEK293 cells. By analyzing the size-exclusion fractions of the media, we found that seed-competent tau was enriched in high molecular weight fractions of >2,000 kDa, while the majority of soluble tau in the media positively detected by ELISA was in low molecular weight fractions. We also found that lysosomal stress not only increased Ca^2+^-dependent release of seed-competent tau but also altered its molecular size. Inhibiting lysosomal exocytosis specifically decreased release of seed-competent tau without influencing total tau. These data underscore the differential response of seed-competent tau and inert tau to lysosomal stress and indicates the presence of distinct release mechanisms via lysosomes.

## Introduction

Tau pathology manifests in synaptically connected brain regions in Alzheimer’s disease (Braak and Braak, 1991). *Trans*-cellular propagation has been considered as one of the mechanisms that could account for the spatiotemporal progression of tau pathology (Clavaguera et al., 2009; Frost et al., 2009). This process is initiated by the release of a certain form of tau into the extracellular space, followed by its uptake by recipient cells and subsequent seed-dependent aggregation.

Over the last few years, there has been considerable interests in identifying specific tau species involved in this process (herein referred to as seed-competent tau); nonetheless, the molecular characteristics of seed-competent tau remained controversial. Earlier studies have shown that insoluble, fibrillar forms of tau can spontaneously bind to cell surface and trigger the uptake (Frost et al., 2009; Santa-Maria et al., 2012). However, even soluble tau, especially phosphorylated tau with high molecular weight has been suggested to be efficiently taken up by neurons (Takeda et al., 2015). Moreover, recent studies have revealed that much smaller tau species including low molecular weight oligomers or even monomer can be a seed-competent form (Wu et al., 2013; Mirbaha et al., 2015, 2018).

While these studies have collectively indicated that multiple forms of tau could participate in the transmission of tau pathology, the findings are based only on seeding ability of recombinant tau or tau in brain lysates that mostly represent its intracellular form. Therefore, a critical question remains as to what forms of seed-competent tau are actually released into the extracellular space.

Another unsolved question in the field relates to the release mechanism of seed-competent tau. Despite being a cytoplasmic protein, tau is also physiologically released into the extracellular space in response to various external stimuli (Yamada et al., 2011, 2014; Karch et al., 2012; Pooler et al., 2013). It remains unknown whether the release of seed-competent tau is also facilitated by external stimuli and whether the release occurs via the same pathway as normal soluble tau.

In this study, by discriminating seed-competent tau from inert soluble tau using tau biosensor cells expressing P301S mutant tau fused to CFP/YFP (Holmes et al., 2014), we sought to re-characterize the molecular nature of extracellular seed-competent tau and investigate the mechanisms that specifically influence release of seed-competent tau.

## Materials and Methods

### Plasmids

2N4R human tau and tau repeat domain (RD) in pNG2 were kind gifts from Dr. E.-M. Mandelkow. P301S mutation was introduced to RD in pNG2 (a kind gift from Dr. E.-M. Mandelkow) or 1N4R human tau in pcDNA3 (a kind gift from Dr. M. Hasegawa) by site-directed mutagenesis.

### Preparation of Recombinant Tau Fibrils

Recombinant tau was purified as previously described (Aoyagi et al., 2007). RD/P301S tau proteins were fibrillized as previously described (Takahashi et al., 2015) and sonicated using SONIFER 250 (BRANSON).

### HEK293 Cell Culture and Fibril Transduction

HEK293 cells cultured on 6 well plates were transfected with 1N4R P301S mutant tau in pcDNA3 using Lipofectamine 3000 (Life Technologies) or FuGENE6 (Promega). Twenty-four hours later, the cells were treated with 5 μg of RD/P301S fibrils using Lipofectamine 3000 or Multifectam (Promega) for 6 h and the cells were washed with DMEM to remove RD. Media were collected at 72 h after RD transduction and centrifuged at 2,000 × *g* for 10 min and stored at −80°C with the complete protease inhibitors (Roche) and PhosSTOP phosphatase inhibitors (Roche). For immunocytochemistry, cells were plated on PDL-coated cover slips in 12 well plates, and the amounts of reagents and fibrils were reduced into 40%.

### Immunocytochemistry

Cells were fixed with 4% PFA and permeabilized and blocked with 3% BSA solution containing 0.2% Triton X-100. Cells were incubated with indicated primary antibodies (HJ8.5, a kind gift from Dr. Holtzman and PHF-1, a kind gift from Dr. Davies), followed by Alexa fluor 488-conjugated secondary antibodies with DRAQ5.

### ELISA

Tau-5 antibody (0.5 μg/well) was coated in wells of 96 half well plates. On the next day, the plates were blocked with 4% BSA/PBS for 1 h at 37°C. Samples and standards diluted with ELISA sample buffer (0.25% BSA/PBS supplemented with complete) were applied and incubated at 4°C overnight. On the next day, biotinylated HJ8.7 (a kind gift from Dr. Holtzman) or biotinylated BT2 (Thermo Fisher Scientific) were applied at the concentration of 0.3 μg/ml and incubated at 37°C for 1.5 h, followed by incubation with streptavidin-poly-HRP40 (Fitzgerald) at room temperature for 1.5 h. Assays were developed with TMB superslow (Sigma) and the reaction was stopped by addition of 1M H_3_PO_4_ and read at 450 nm.

### Size Exclusion Chromatography

The media were centrifuged at 20,400 × *g* for 5 min and filtered through 30 kDa Amicon ultra and washed 13 times with PBS to eliminate the compounds. The media were then separated by a Superose six column (GE healthcare) in PBS at 0.5 ml/min with an AKTA explorer 10S (GE healthcare). HEK293 cells expressing P301S tau with RD transduction were sonicated with ice cold PBS supplemented with complete and PhosSTOP. After centrifugation at 100,000 × *g* for 20 min, the supernatants were collected as PBS-soluble cell extracts and loaded onto the column. Recombinant 2N4R tau (2.1 μg) was loaded as a control. HMW calibration kit (GE healthcare) was used to estimate molecular weights.

### Detection of Seed-Competent Tau Using Biosensor Cells

The media from HEK293 cells were filtered through 30 kDa Amicon ultra and washed 13 times with PBS to eliminate the compounds. Biosensor cells (ATCC^®^ CRL-3275^TM^) are HEK293 cell line that stably expresses the tau RD with the disease-associated P301S mutation fused to either CFP or YFP (Holmes et al., 2014). Biosensor cells were plated on PDL-coated 12-mm round coverslips in 12 well plate at 1.0 × 10^5^ cells/well. Next day, the filtered media or size exclusion fractions were applied. Seventy-two hours later, biosensor cells were fixed with 4% PFA and the nuclei were counterstained with DRAQ5. The images were obtained via FRET channel (excited with a 458 nm laser and fluorescence was captured with 500–550 nm filter) (Takeda et al., 2016) and FRET positive cell number were counted.

### Immunodepletion

rProtein G agarose beads (Life Technologies) were incubated with indicated antibodies for 4 h with rotation at 4°C. Beads-antibody complex was precipitated, washed three times and incubated with media overnight with rotation at 4°C. Beads-antibody complex was centrifuged and washed three times with PBS. Seeding activity in the supernatant was determined using biosensor cells.

### Statistical Analysis

Statistical analysis was performed using Prism 8 (Graph Pad software). The comparison of two groups was done by unpaired *t*-test. The comparison of multiple groups was done by one-way ANOVA or two-way ANOVA with Tukey’s *post hoc* test. Data are shown as mean ± SEM.

## Results

### Donor HEK293 Cells Release Seed-Competent Tau in High Molecular Weight Fractions of >2,000 kDa

**FIGURE 1:**
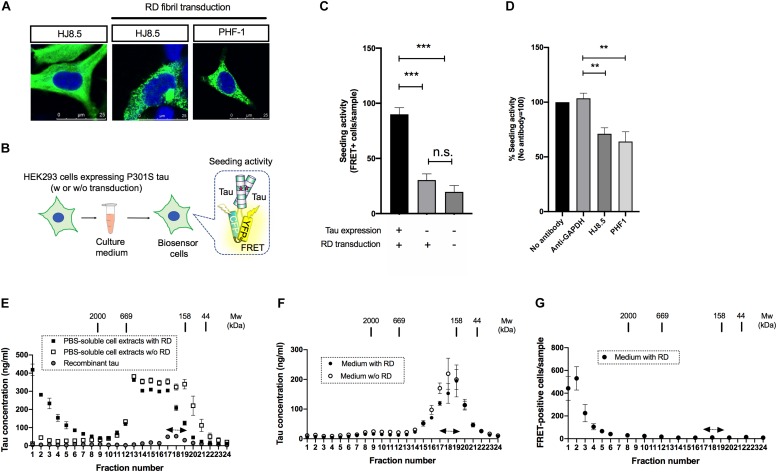
Seed-competent tau released from cells is enriched in the fractions of ∼2,000kDa. **(A)** HEK293 cells expressing full-length tau with P301S mutation were immunostained with HJ8.5 antibody or PHF-1 antibody (green) after transduction of RD fibrils. Nuclei were stained by DRAQ5 (blue). **(B)** Schematic depiction of tau propagation assay used in the study. **(C)** The addition of media from P301S full-length tau expressing HEK293 cells transduced with RD fibrils significantly increased FRET-positive biosensor cells compared to the cells without tau expression or the cells without tau expression and RD transduction. *N* = 3, ^∗∗∗^*p* < 0.001. **(D)** The media from full-length tau expressing cells with RD transduction were incubated by indicated antibodies. Seeding activities in immunodepleted media were analyzed by tau biosensor cells. *N* = 4. ^∗∗^*p* < 0.01. **(E)** The PBS-soluble cell extracts from HEK293 cells expressing full-length tau with or without RD transduction were separated using a size-exclusion chromatography. *N* = 3. Eluted fractions were analyzed with Tau-5/HJ8.7 ELISA. Recombinant 2N4R tau was loaded as a control. Monomer fractions predicted by recombinant tau were indicated by arrows. **(F)** The media from full-length tau expressing cells with or without RD transduction were separated using a size-exclusion chromatography. Eluted fractions were analyzed with Tau-5/HJ8.7 ELISA. *N* = 4. **(G)** The media from full-length tau expressing cells with RD transduction were separated by a size-exclusion chromatography and analyzed with tau biosensor cells. *N* = 3.

To characterize seed-competent tau released into the media, we first prepared donor cells harboring tau aggregates. To this end, HEK293 cells expressing full-length form of tau with P301S mutation were transduced with fibrils made of tau RD harboring P301S mutation. Six hours after transduction, the fibrils were removed and the cells were analyzed at 3-day post transduction using two different tau antibodies (i.e., HJ8.5 and PHF-1) that do not recognize RD. Full-length tau was diffusely distributed in the cytosol without RD transduction, whereas it formed aggregates in discrete puncta upon RD transduction, consistent with the previous studies (Frost et al., 2009) (Figure 1A).

Biosensor cells stably express two forms of RD with P301S mutation fused to CFP/YFP and thereby accumulate FRET-positive aggregates upon the entry of seed-competent tau (Holmes et al., 2014). To specifically evaluate extracellular seed-competent tau that can spontaneously enter the cells, we collected media from the donor HEK293 cells and applied them to the recipient biosensor cells without lipofection that forces internalization of tau (Figure 1B). Addition of the media from cells expressing P301S full length tau with RD transduction resulted in a robust increase of FRET-positive biosensor cells (Figure 1C). In contrast, the media from cells without full-length tau expression yielded significantly less FRET-positive biosensor cells, which were at similar levels to that from cells without both tau expression and RD transduction (Figure 1C). The result suggests that the robust seeding activity in donor HEK293 media requires both full-length tau expression and RD transduction.

To see whether seeding activity is mediated by tau expressed in donor HEK293 cells, we have also performed immunodepletion assays using anti-tau antibodies that do not recognize RD. Addition of media after immunodepletion with both HJ8.5 (recognizing amino acids 7–13) and PHF-1 (recognizing phosphorylated amino acids at 396 and 404) antibodies significantly reduced FRET-positive biosensor cells compared to GAPDH antibody used as a control, confirming that seeding activity is not due to potentially remaining RD fibrils (Figure 1D).

Next, we sought to assess the molecular weight of seed-competent tau by a size exclusion chromatography. We first fractionated PBS-soluble cell extracts of HEK293 cells transduced with or without RD and analyzed them by ELISA. Cellular tau from cells with RD transduction were distributed in various sizes ranging from >2,000 kDa fractions to monomer fractions (fraction No. 17–19, indicated by the arrows) whose molecular weight match that of full-length recombinant tau loaded separately as a control (Figure 1E). Tau levels from cells without RD transduction were mainly distributed in fractions No. 13–19 whose molecular weights correspond to 158–669kDa. These results suggest that intracellular tau forms high molecular weight species upon RD transduction. In contrast, when the media was fractionated, ELISA showed the most robust signals at monomer fractions and only a trace amount of signals at fractions >2,000 kDa regardless of RD transduction (Figure 1F). These data indicate that release efficiency depends on tau species: with high efficiency for lower molecular weight tau and low efficiency for high molecular weight tau.

Next we analyzed these size exclusion fractions of the media from cells with RD transduction with biosensor cells. The largest number of FRET-positive biosensor cells were observed upon exposure to fraction No. 1–3 (>2,000 kDa), while only a few positive cells were yielded by addition of the presumable monomeric fractions (No. 17–19) that exhibited the most robust ELISA signals (Figure 1F). This result suggests that extracellular, seed-competent tau exists as >2,000 kDa high molecular weight species, whose levels in the media are markedly low in abundance yet specifically detected by biosensor cells.

### Lysosomal Stress Increased the Release of Seed-Competent Tau in a Ca^2+^-Dependent Manner

**FIGURE 2:**
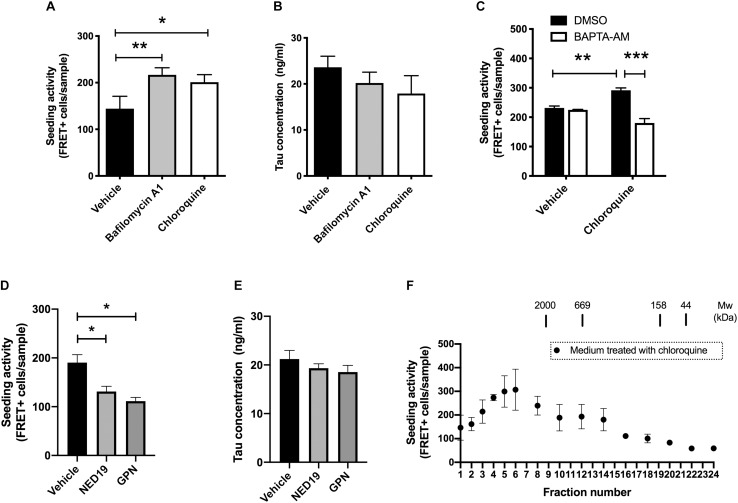
Lysosomal stress increases the release of seed-competent tau. **(A)** Full-length tau expressing cells with RD transduction were treated with bafilomycin A1 (100 nM) or chloroquine (100 μM) for 2 h. The seeding activities in the media were analyzed with biosensor cells. *N* = 3–6. ^∗^*p* < 0.05, ^∗∗^*p* < 0.01. **(B)** Tau concentration in media detected by Tau-5/BT2 ELISA was not altered by treatment with bafilomycin A1 or chloroquine. *N* = 3–6. **(C)** Pre-treatment with BAPTA-AM (20 μM) for 30 min significantly inhibited the increase of FRET-positive biosensor cells by chloroquine. *N* = 3, ^∗∗^*p* < 0.01, ^∗∗∗^*p* < 0.001. **(D)** The addition of media from cells treated with GPN (200 μM) or NED-19 (10 μM) significantly decreased the number of FRET-positive biosensor cells. *N* = 3, ^∗^*p* < 0.05. **(E)** Tau concentration in media detected by Tau-5/HJ8.7 ELISA was not altered by treatment with GPN or NED-19. *N* = 3. **(F)** Full-length tau expressing cells with RD transduction were treated with chloroquine for 2 h. The media were separated with a size-exclusion chromatography and analyzed with tau biosensor cells. *N* = 3.

The amount of normal tau released from cells are influenced by various external stimuli (Pooler et al., 2013; Mohamed et al., 2014; Yamada et al., 2014). This led us to hypothesize that the release of seed-competent tau is also altered upon stimulation. Because the dysregulation of lysosomes has been implicated in the transmission of aggregated proteins (Victoria and Zurzolo, 2017), we examined if lysosomal stress influences its release. We briefly exposed donor HEK293 cells transduced with RD to bafilomycin A1 or chloroquine, which differentially impairs lysosomal functions by neutralizing pH or accumulating in its lumen, respectively. Although prolonged treatment with bafilomycin A1 or chloroquine has been shown to increase cellular tau levels by inhibiting its lysosomal degradation (Jo et al., 2014), 2-h exposure of these compounds did not cause detectable changes in the cellular tau levels (data not shown). Nonetheless, the addition of the media from cells treated with bafilomycin A1 or chloroquine resulted in a significant increase in the number of FRET-positive biosensor cells (Figure 2A). In contrast, both treatments did not change the levels of inert soluble tau in media detected by ELISA (Figure 2B). Interestingly, when BAPTA-AM, a membrane-permeable Ca^2+^ chelator, was administered prior to chloroquine treatment, the addition of the media did not increase the number of FRET-positive biosensor cells (Figure 2C).

Both chloroquine and bafilomycin A1 are known to enhance lysosomal exocytosis, which is a Ca^2+^ dependent exocytosis process (Eguchi et al., 2018; Tsunemi et al., 2019). Therefore, we hypothesized that lysosomal exocytosis is involved in release of seed-competent tau. To test this hypothesis, we inhibited lysosomal exocytosis by two different compounds, Glycyl-L-phenylalanine 2-naphthylamide (GPN) and NED-19. GPN is a specific substrate of cathepsin C and disrupts lysosomal membrane by accumulating inside of lysosomes (Ibata et al., 2019). In contrast, NED-19 is an antagonist of NAADP, which is required for Ca^2+^ release from lysosomes (Padamsey et al., 2017). Addition of the media from cells treated with GPN or NED-19 resulted in a significant decrease in FRET-positive biosensor cells without altering tau concentration detected by ELISA (Figures 2D,E). Taken together, these data suggest that lysosomal stress exerts a unique mechanism of release on seed-competent tau, but not on inert soluble tau likely via lysosomal exocytosis.

Next, we asked whether the size of seed-competent tau is changed upon chloroquine treatment. When the media from cells treated with chloroquine were separated by size exclusion chromatography, we found that the distribution of seeding activity was distinct from those in cells without chloroquine treatment. While the media from cells without chloroquine had a peak of seeding activity in fractions No. 1–3 (Figure 1G), chloroquine shifted the peak toward smaller molecular weights (fractions No. 4–6) (Figure 2F). The data suggest that chloroquine treatment also alters the size of seed-competent tau released from cells.

## Discussion

While multiple forms of tau with various sizes ranging from fibrils to a monomer formed *in vitro* have previously been shown to have a seeding activity, the molecular characteristics of seed-competent tau released from cells has remained to be elucidated. Utilizing biosensor cells to specifically detect seed-competent tau, we found that tau species with a molecular weight of >2,000 kDa present in media spontaneously enter the biosensor cells without the aid of lipofection and trigger seed-dependent aggregation. While seeding activity was only evident in the media from cells with RD transduction, ELISA detected very low levels of tau with a molecular weight of >2,000 kDa regardless of RD transduction. This data indicate that tau might form high molecular weight complexes that do not have seeding activity, which can be discriminated by biosensor cells.

We also found that lysosomal stress caused by bafilomycin A1 and chloroquine enhanced the release of seed-competent tau without altering release of inert soluble tau. Intriguingly, the increased release of seed-competent tau by chloroquine was mediated in a Ca^2+^-dependent manner, whereas release of inert soluble tau was not affected by chloroquine.

The dysregulation of lysosomes has been implicated in the transmission of aggregated proteins, although how it will impact tau propagation has not been empirically tested. In this study, we have first demonstrated the involvement of lysosomal exocytosis on release of seed-competent tau by using GPN and NED-19. These data collectively indicate that the release of seed-competent tau and inert soluble tau are differentially regulated under lysosomal stresses and by lysosomal exocytosis.

The seeding activity in the media was not completely abolished by GPN and NED-19, suggesting that other secretory pathways might be also involved. For example, tau in exosomes has been shown to propagate between cells (Wang et al., 2017), which is consistent with partial inhibition of seeing activity by antibodies in our study.

By analyzing the media with size exclusion chromatography, we also found that chloroquine treatment increased seed-competent tau of smaller molecular weight ranges than control. This could be due to the processing that occurs during lysosomal exocytosis. Given that shorter fibrils are more potent to induce aggregation than longer fibrils (Tarutani et al., 2016), seed-competent tau with smaller molecular weights increased by chloroquine might be more potent to propagate between cells.

Although HEK293 propagation models were used in this study as they release significant amount of seed-competent tau and thus allows its better quantification, neuronal models will be necessary to further investigate how lysosomal exocytosis influences the propagation between synaptically connected neurons as well as whether neurons also show similar uptake preferences toward seed-competent tau with different molecular weight sizes.

In addition, it should also be noted that multiple tau strains have been shown to propagate distinct amyloid conformations (Sanders et al., 2014). Although we detected the seeding activity of tau exclusively in the >2,000 kDa fractions, it would be possible that distinct tau strains form seed-competent tau of different sizes.

Further studies using different types of cells, aggregates or mutations will enable further detailed characterization of the molecular nature of extracellular seed-competent tau.

Given that inert normal tau is also actively released into the extracellular space, therapeutic strategies such as simple sequestration of extracellular tau may interfere with its yet-to-be identified physiological functions. From this perspective, being able to distinguish seed-competent tau from inert soluble tau in this study should help identify novel cellular processes that specifically influence tau propagation without disturbing its normal functions.

## Data Availability Statement

All datasets generated for this study are included in the article/supplementary material.

## Author Contributions

YT and KY conceived the idea with critical input from TI. YT, KY, KS, and TK designed the experiments. YT, KY, KS, IN, and MH performed the experiments. TK and TI assisted with interpreting the results. KY and TI wrote the manuscript with critical input from TK. All authors read and approved the final version of the manuscript.

## Conflict of Interest

The authors declare that the research was conducted in the absence of any commercial or financial relationships that could be construed as a potential conflict of interest.

## References

[B1] Aoyagi H. Hasegawa M. Tamaoka A. (2007). Fibrillogenic nuclei composed of P301L mutant tau induce elongation of P301L tau but not wild-type tau. *J. Biol. Chem.* 282 20309–20318. 10.1074/jbc.M611876200 17526496

[B2] Braak H. Braak E. (1991). Neuropathological stageing of Alzheimer-related changes. *Acta Neuropathol.* 82 239–259. 10.1007/BF00308809 1759558

[B3] Clavaguera F. Bolmont T. Crowther R. A. Abramowski D. Frank S. Probst A. (2009). Transmission and spreading of tauopathy in transgenic mouse brain. *Nat. Cell Biol.* 11 909–913. 10.1038/ncb1901 19503072 PMC2726961

[B4] Eguchi T. Kuwahara T. Sakurai M. Komori T. Fujimoto T. Ito G. (2018). LRRK2 and its substrate Rab GTPases are sequentially targeted onto stressed lysosomes and maintain their homeostasis. *Proc. Natl. Acad. Sci. U.S.A.* 115 E9115–E9124. 10.1073/pnas.1812196115 30209220 PMC6166828

[B5] Frost B. Jacks R. L. Diamond M. I. (2009). Propagation of Tau misfolding from the outside to the inside of a cell. *J. Biol. Chem.* 284 12845–12852. 10.1074/jbc.M808759200 19282288 PMC2676015

[B6] Holmes B. B. Furman J. L. Mahan T. E. Yamasaki T. R. Mirbaha H. Eades W. C. (2014). Proteopathic tau seeding predicts tauopathy in vivo. *Proc. Natl. Acad. Sci. U.S.A.* 111 E4376–E4385. 10.1073/pnas.1411649111 25261551 PMC4205609

[B7] Ibata K. Kono M. Narumi S. Motohashi J. Kakegawa W. Kohda K. (2019). Activity-dependent secretion of synaptic organizer cbln1 from lysosomes in granule cell axons. *Neuron* 102 1184.e10–1198.e10. 10.1016/j.neuron.2019.03.044 31072786

[B8] Jo C. Gundemir S. Pritchard S. Jin Y. N. Rahman I. Johnson G. V. W. (2014). Nrf2 reduces levels of phosphorylated tau protein by inducing autophagy adaptor protein NDP52. *Nat. Commun.* 5 1–13. 10.1038/ncomms4496 24667209 PMC3990284

[B9] Karch C. M. Jeng A. T. Goate A. M. (2012). Extracellular tau levels are influenced by variability in tau that is associated with tauopathies. *J. Biol. Chem.* 287 42751–42762. 10.1074/jbc.M112.380642 23105105 PMC3522274

[B10] Mirbaha H. Chen D. Morazova O. A. Ruff K. M. Sharma A. M. Liu X. (2018). Inert and seed-competent tau monomers suggest structural origins of aggregation. *elife* 7:e36584. 10.7554/eLife.36584 29988016 PMC6039173

[B11] Mirbaha H. Holmes B. B. Sanders D. W. Bieschke J. Diamond M. I. (2015). Tau trimers are the minimal propagation unit spontaneously internalized to seed intracellular aggregation. *J. Biol. Chem.* 290 14893–14903. 10.1074/jbc.M115.652693 25887395 PMC4463437

[B12] Mohamed N.-V. Plouffe V. Rémillard-Labrosse G. Planel E. Leclerc N. (2014). Starvation and inhibition of lysosomal function increased tau secretion by primary cortical neurons. *Sci. Rep.* 4 1–11. 10.1038/srep05715 25030297 PMC4101526

[B13] Padamsey Z. McGuinness L. Bardo S. J. Reinhart M. Tong R. Hedegaard A. (2017). Activity-dependent exocytosis of lysosomes regulates the structural plasticity of dendritic spines. *Neuron* 93 132–146. 10.1016/j.neuron.2016.11.013 27989455 PMC5222721

[B14] Pooler A. M. Phillips E. C. Lau D. H. W. Noble W. Hanger D. P. (2013). Physiological release of endogenous tau is stimulated by neuronal activity. *EMBO Rep.* 14 389–394. 10.1038/embor.2013.15 23412472 PMC3615658

[B15] Sanders D. W. Kaufman S. K. DeVos S. L. Sharma A. M. Mirbaha H. Li A. (2014). Distinct tau prion strains propagate in cells and mice and define different tauopathies. *Neuron* 82 1271–1288. 10.1016/j.neuron.2014.04.047 24857020 PMC4171396

[B16] Santa-Maria I. Varghese M. Ksiezak-Reding H. Dzhun A. Wang J. Pasinetti G. M. (2012). Paired helical filaments from alzheimer disease brain induce intracellular accumulation of Tau Protein in aggresomes. *J. Biol. Chem.* 287 20522–20533. 10.1074/jbc.M111.323279 22496370 PMC3370237

[B17] Takahashi M. Miyata H. Kametani F. Nonaka T. Akiyama H. Hisanaga S. (2015). Extracellular association of APP and tau fibrils induces intracellular aggregate formation of tau. *Acta Neuropathol.* 129 895–907. 10.1007/s00401-015-1415-2 25869641 PMC4436700

[B18] Takeda S. Commins C. DeVos S. L. Nobuhara C. K. Wegmann S. Roe A. D. (2016). Seed-competent high-molecular-weight tau species accumulates in the cerebrospinal fluid of Alzheimer’s disease mouse model and human patients. *Ann. Neurol.* 80 355–367. 10.1002/ana.24716 27351289 PMC5016222

[B19] Takeda S. Wegmann S. Cho H. DeVos S. L. Commins C. Roe A. D. (2015). Neuronal uptake and propagation of a rare phosphorylated high-molecular-weight tau derived from Alzheimer’s disease brain. *Nat. Commun.* 6:8490. 10.1038/ncomms9490 26458742 PMC4608380

[B20] Tarutani A. Suzuki G. Shimozawa A. Nonaka T. Akiyama H. Hisanaga S. I. (2016). The effect of fragmented pathogenic α-synuclein seeds on prion-like propagation. *J. Biol. Chem.* 291 18675–18688. 10.1074/jbc.M116.734707 27382062 PMC5009244

[B21] Tsunemi T. Perez-Rosello T. Ishiguro Y. Yoroisaka A. Jeon S. Hamada K. (2019). Increased lysosomal exocytosis induced by lysosomal Ca ^2+^ channel agonists protects human dopaminergic neurons from α-synuclein toxicity. *J. Neurosci.* 39 5760–5772. 10.1523/jneurosci.3085-18.2019 31097622 PMC6636071

[B22] Victoria G. S. Zurzolo C. (2017). The spread of prion-like proteins by lysosomes and tunneling nanotubes: implications for neurodegenerative diseases. *J. Cell Biol.* 216 2633–2644. 10.1083/jcb.201701047 28724527 PMC5584166

[B23] Wang Y. Balaji V. Kaniyappan S. Krüger L. Irsen S. Tepper K. (2017). The release and trans-synaptic transmission of Tau via exosomes. *Mol. Neurodegener.* 12:5. 10.1186/s13024-016-0143-y 28086931 PMC5237256

[B24] Wu J. W. Herman M. Liu L. Simoes S. Acker C. M. Figueroa H. (2013). Small misfolded Tau species are internalized via bulk endocytosis and anterogradely and retrogradely transported in neurons. *J. Biol. Chem.* 288 1856–1870. 10.1074/jbc.M112.394528 23188818 PMC3548495

[B25] Yamada K. Cirrito J. R. Stewart F. R. Jiang H. Finn M. B. Holmes B. B. (2011). In vivo microdialysis reveals age-dependent decrease of brain interstitial fluid Tau Levels in P301S human tau transgenic mice. *J. Neurosci.* 31 13110–13117. 10.1523/JNEUROSCI.2569-11.2011 21917794 PMC4299126

[B26] Yamada K. Holth J. K. Liao F. Stewart F. R. Mahan T. E. Jiang H. (2014). Neuronal activity regulates extracellular tau in vivo. *J. Exp. Med.* 211 387–393. 10.1084/jem.20131685 24534188 PMC3949564

